# Presence of zoonotic agents in engorged ticks and hedgehog faeces from *Erinaceus europaeus* in (sub) urban areas

**DOI:** 10.1186/s13071-015-0814-5

**Published:** 2015-04-09

**Authors:** Aleksandra I Krawczyk, Arieke Docters van Leeuwen, Wilma Jacobs-Reitsma, Lucas M Wijnands, El Bouw, Setareh Jahfari, Angela H A M van Hoek, Joke W B van der Giessen, Jeroen H Roelfsema, Michiel Kroes, Jenny Kleve, Yolanda Dullemont, Hein Sprong, Arnout de Bruin

**Affiliations:** Centre for Zoonoses & Environmental Microbiology, Centre for Infectious Disease Control, National Institute for Public Health and the Environment, Bilthoven, the Netherlands; Centre for Research Infectious Diseases Diagnostics and Screening, Centre for Infectious Disease Control, National Institute for Public Health and the Environment, Bilthoven, the Netherlands; Stichting Egelbescherming Nederland, Naarden, the Netherlands

**Keywords:** Hedgehogs, Ticks, Zoonoses, *Borrelia*, *Anaplasma*, *Campylobacter*, *Salmonella*, Antibiotic resistance, *Giardia*, *Cryptosporidium*

## Abstract

**Background:**

European hedgehogs (*Erinaceus europaeus*) are hosts for *Ixodes hexagonus* and *I. ricinus* ticks, which are vectors for zoonotic microorganisms. In addition, hedgehogs may carry several enteric zoonoses as well. It is unclear to what extent a presence of pathogens in hedgehogs poses a risk to public health, as information on the presence of zoonotic agents in hedgehogs in urban areas is relatively scarce.

**Methods:**

Engorged ticks and hedgehog faeces were collected from rehabilitating hedgehogs. Ticks were screened individually for presence of *Borrelia burgdorferi* sensu lato, *B. miyamotoi*, *Anaplasma phagocytophilum*, and *Candidatus* Neoehrlichia mikurensis using PCR-based assays. Faecal samples were screened for presence of *Campylobacter, Salmonella*, *Giardia, Cryptosporidium*, and extended-spectrum cephalosporin-resistant-*Escherichia coli* (ESC)-resistant *E. coli*, using both culture-based and PCR-based methods.

**Results:**

*Anaplasma phagocytophilum* and *Borrelia* genospecies *B. afzelii*, *B. spielmanii*, *B. garinii*, and *B. burgdorferi* sensu stricto were detected in both *I. hexagonus* and *I. ricinus* ticks. Despite their widespread distribution in the Netherlands, *B. miyamotoi* and *Candidatus* N. mikurensis were not detected in collected ticks. Analysis of hedgehog faecal samples revealed the presence of *Salmonella enterica* subspecies *enterica* and *Campylobacter jejuni*. In addition, ESC-resistant *E. coli* were observed in high prevalence in faecal samples, but no Shiga-toxin producing*-E.coli* were detected. Finally, potentially zoonotic protozoan parasites were observed in hedgehog faecal samples as well, including *Giardia duodenalis* assemblage A, *Cryptosporidium parvum* subtypes IIaA17G1R1 and IIcA5G3, and *C. hominis* subtype IbA10G2.

**Conclusions:**

European hedgehogs in (sub)urban areas harbor a number of zoonotic agents, and therefore may contribute to the spread and transmission of zoonotic diseases. The relatively high prevalence of *B. burgdorferi* s.l. and *A. phagocytophilum* in engorged ticks, suggests that hedgehogs contribute to their enzootic cycles in (sub)urban areas. To what extent can hedgehogs maintain the enteric zoonotic agents in natural cycles, and the role of (spill-back from) humans remains to be investigated.

## Background

Hedgehogs are host to a wide variety of bacterial and protozoan pathogens [1-3], of which a number have become a matter of concern to public health. Since hedgehogs often dwell in (sub)urban areas, people who rescue or rehabilitate hedgehogs can be exposed to a variety of these pathogens by contact with hedgehogs, their excrements, and vectors. European hedgehogs (*Erinaceus europaeus*) are a reservoir host for *Borrelia burgdorferi* sensu lato (Lyme borreliosis), which is widely distributed in the Netherlands, and contributes to maintenance of the bacterium in an enzootic cycle [3,4]. In addition, it was proposed that European hedgehogs are a suitable reservoir host for *Anaplasma phagocytophilum*, which causes granulocytic anaplasmosis in humans [2]. Both *Borrelia* genospecies and *A. phagocytophilum* are transmitted by ixodid ticks, such as *Ixodes ricinus* that feed on various hosts and *I. hexagonus* that feed predominantly on European hedgehogs [5]. All three life stages of these tick species can feed on humans [6].

In addition to vector-borne agents, hedgehogs are a potential reservoir for enteric bacteria (such as *Salmonella* and *Campylobacter*), and protozoan parasites (*Giardia* and *Cryptosporidium*), which may cause enteritis in humans, livestock, and pets [1,7-9]. The primary transmission route to humans is believed to be food-borne, however, (indirect) contact with an animal reservoir can be an alternative source of infection [9,10]. For instance, a study carried out in Denmark reported that strains of *Salmonella* Enteritidis, isolated from European hedgehogs, belong to the same clonal lineage as strains isolated from infected humans [11].

In contrast, the zoonotic potential of some enteric protozoan parasites has not been fully recognized. Many studies designed to determine genetic groups of protozoan parasites in various hosts, suggest a limited zoonotic potential for *Giardia*, since strains isolated from people were infrequently found in animals [12,13]. Although zoonotic transmission of livestock-associated *Cryptosporidium* has frequently been described [14], the extent to which wildlife (e.g. hedgehogs) act as a source for *Cryptosporidium* infection in humans remains unclear.

Finally, little is known about the potential reservoir competence of the European hedgehog for other pathogens transmitted by ixodid ticks, such as *Candidatus* Neoehrlichia mikurensis, an agent of human neoehrlichiosis, and *B. miyamotoi*, a recently discovered agent belonging to the relapsing fever group. A number of studies detected *Candidatus* N. mikurensis in Northern white-breasted hedgehog (*Erinaceus roumanicus*) tissue samples in Hungary, and in *I. hexagonus* feeding on hedgehogs and dogs in the Netherlands and Germany, respectively [15-17]. However, the role of European hedgehogs and their ectoparasites in maintenance of this pathogen in an enzootic cycle is unknown. *Borrelia miyamotoi* is present in questing *I. ricinus* in the Netherlands [18], however, it has never been investigated in *I. hexagonus* before*.*

In the current study, the presence of a number of zoonotic vector-borne and enteric bacteria and two protozoan parasites was investigated in engorged ticks, obtained from European hedgehogs and hedgehog faeces. In addition, the presence of extended-spectrum cephalosporin (ESC)-resistant *E. coli* was investigated in faeces, since ESC-resistant *E. coli* are found in many animal and environmental reservoirs.

## Methods

### Collection of *Ixodes* ticks and DNA extraction procedures

*Ixodes hexagonus* and *I. ricinus* ticks were collected from European hedgehogs, rehabilitating in a hedgehog shelter in the city of Naarden, and obtained via the Dutch Wildlife Health Centre (DWHC, Utrecht) in 2010, 2011, and 2012. All hedgehogs originated from five different provinces in the Netherlands: Flevoland, Gelderland, Noord-Holland, Utrecht, and Zuid-Holland. In addition, 15 *I. hexagonus* ticks were collected from dead hedgehogs near the city of Ede (province of Gelderland), and from a zoo in the city of Emmen (province of Drenthe) in 2014. Collected samples included ticks of both sexes and all developmental stages with a majority of adult female ticks.

DNA from partially engorged ticks was extracted with ammonium hydroxide as described previously [19]. DNA from fully engorged ticks was extracted using the Qiagen DNeasy Blood & Tissue Kit according to the manufacturer’s protocol for the purification of total DNA from ticks (Qiagen, Venlo, the Netherlands).

### Detection of tick-borne pathogens using qPCR, conventional PCR, and sequencing procedures

Ticks were tested individually for presence of *B. burgdorferi* s.l., *B. miyamotoi*, *A. phagocytophilum* and *Candidatus* N. mikurensis using (q)PCR assays, followed by sequencing for species identification when necessary. For the detection of *B. burgdorferi* s.l., a duplex real-time PCR was used, based on the detection of fragments of *ospA* and flagellin genes [20]. A conventional PCR assay, targeting the 5S-23S intergenic region [(IGS) was performed, for *Borrelia* genospecies identification [21]. Both strands of PCR products were sequenced by BaseClear (Leiden, The Netherlands), using the same forward and reverse primers as in conventional PCR. *Borrelia* genospecies identification was determined by comparison of sequences to isolates in-house molecular databases (PMID: 23602839). For detection of *B. miyamotoi*, a real-time PCR assay was used that targets a region of the flagellin gene, specific for *B. miyamotoi* [18]. For detection of *A. phagocytophilum* and *Candidatus* N. mikurensis a single duplex real-time PCR assay was used that targets a region of the *A. phagocytophilum* major surface protein (*msp2*) gene [22], and a region specific for *Candidatus* N. mikurensis of the heat shock protein gene *groEL* [17]. Due to limitations of available DNA, not all ticks (n = 628) were tested for all pathogens. For numbers of ticks tested for each vector-borne pathogen, see Table 1.

**Table 1:** **Prevalence of tick-borne pathogens in ticks feeding on hedgehogs**

	***Ixodes hexagonus***		***Ixodes ricinus***	
	**n**	**%**	**n**	**%**
*B. burgdorferi *s.l.	60/435	14	7/25	28
*B. afzelii*	37/49	76	n.d.	-
*B. garinii*	3/49	6	n.d.	-
*B. spielmani*	7/49	14	n.d.	-
*B. burgdorferi s.s.*	2/49	4	n.d.	-
*B. miyamotoi*	0/170	0	0/25	0
A. phagocytophilum*	74/277	27	6/25	24
*Candidatus* N. mikurensis**	0/251	0	0/25	0

n.d. = not determined.

*Our results (30/84) were compiled with data obtained from [40], where 44/193 *I. hexagonus* ticks were found positive for *A. phagocytophilum.*

**Our results (0/84) were compiled with data from [17], where none of the 167 tested *I. hexagonus* ticks were found positive for *Candidatus* N. mikurensis.

### Collection of hedgehog faeces, DNA extraction, detection of enteric pathogens, protozoan parasites, and anti-microbial resistance genes

No ethical approval is required for the experimental methods used in this study. The hedgehog shelter has a permit for handling and rehabilitating hedgehogs by the State Secretary for Economic Affairs, Agriculture and Innovation, according article 75 of the Dutch ‘Animal Health and Welfare Act’. Hedgehog faeces were collected from 90 hedgehogs, rehabilitating in the hedgehog shelter in the city of Naarden in April (n = 58) and October (n = 32) of 2013. Hedgehogs originated from five different provinces in the Netherlands, described before, and were brought to the shelter due to apparent sickness or injury. Hedgehog faecal material was examined for the presence of *Campylobacter* by standard microbiological methods, according to ISO/DIS 10272–1 [23]. Confirmation was based on typical microscopic appearance of suspect colonies on mCCDA plates, and by PCR in order to distinguish between *C. coli*, *C. jejuni*, *C. lari* and *C. upsaliensis* isolates [24].

Faecal samples were also tested for the presence of *Salmonella* according to Annex D of ISO 6579 [25], and Shiga toxin-producing *E. coli* according to ISO/TS 13136 [26]. After the presence of *Salmonella* was confirmed, serotyping was performed using the method of Grimont and Weill [27].

Expanded spectrum cephalosporin-resistant *E. coli* (ESC-resistant *E. coli*) were isolated by direct streaking of a loop (10 μl) of hedgehog faeces on Brilliance *E. coli*/coliform Selective Agar (Oxoid), supplemented with 1 μg/ml cefotaxime (Sigma). Suspected ESC-resistant *E. coli* were phenotypically confirmed with a combination disc-diffusion test according to CLSI guidelines [28]. Cefotaxime and ceftazidime discs, with and without clavulanic acid, were used to identify ESBL-producing *E. coli*. A cefoxitin disc was used to detect isolates with an AmpC phenotype.

For detection of protozoan parasites, DNA was isolated from faecal samples using the High Pure PCR template DNA isolation kit from Roche (Almere, The Netherlands), according to the manufacturer’s instructions. Detection of *Giardia duodenalis, Cryptosporidium parvum,* and *C. hominis* was performed using a multiplex real-time PCR [29]. Molecular typing of *Cryptosporidium* species was performed by sequencing an amplified fragment of the GP60 gene [30]. The assemblage of *G. duodenalis* was established using a PCR on marker 4E1-HP, specific for either assemblage A or B [31], which are associated with human infections.

## Results and discussion

Regarding tick-borne pathogens, we detected *B. burgdorferi* s.l. in 14% (60/435) of *I. hexagonus* ticks and 28% (7/25) of *I. ricinus* ticks feeding on European hedgehogs (Table 1). Intergenic spacer (IGS) sequencing of 49 PCR-positive *I. hexagonus* ticks revealed several known *Borrelia* genospecies: *B. afzelii* (76%), *B. spielmanii* (14%), *B. garinii* (6%), and *B. burgdorferi* s.s. (4%; Table 1). These findings are consistent with previous studies, which revealed the presence of the same *Borrelia* genospecies in ticks feeding on hedgehogs in Germany and Switzerland [3,4]. This suggests that the European hedgehog may be a reservoir host for *B. burgdorferi* s.l. also in the Netherlands as, and may influence local Lyme borreliosis risk.

In addition to *B. burgdorferi* s.l. genospecies, *A. phagocytophilum* was detected as well in *Ixodes* ticks feeding on European hedgehogs. DNA was detected in 27% (68/251) of *I. hexagonus* ticks and in 24% (6/25) of *I. ricinus* ticks (Table 1). The relatively high prevalence of *A. phagocytophilum* found in the current study supports the idea, proposed by other researchers, that *E. europaeus* is a reservoir host for this pathogen [2,4,32].

*Ixodes hexagonus* as a nidicolous species, rarely bites humans and its direct epidemiological importance is unknown [5,6]. However, it seems to contribute to the circulation of both *B. burgdorferi* s.l., and *A. phagocytophilum* in nature [2,33]. In addition its predominant host, *E. europaeu*s, may harbour all life stages of generalist *I. ricinus* ticks, which successfully infect hedgehogs with at least one major group of zoonotic agents: *B. burgdorferi* s.l. [34]. In certain habitats, hedgehogs may be the main host for *I. ricinus* ticks, which may acquire pathogens via either co-feeding or systemic transmission [35]. Subsequently, *I. ricinus* ticks may transmit a number of bacterial pathogens (e.g. *A. phagocytophilum* and *B. burgdorfei* s.l.) to other vertebrates as well, including humans.

To our knowledge, this is the first study that has tested ticks feeding on hedgehogs for *B. miyamotoi,* a spirochete belonging to the relapsing fever group. The absence of this pathogen in *I. hexagonus* may indicate that this specialist tick species is not a competent vector, or that *E. europaeus* is not a competent host for *B. miyamotoi.* However, it was shown that 4% of questing *I. ricinus* ticks were positive for *B. miyamotoi* in the Netherlands [36]. Therefore, it is also possible that the number of investigated *I. ricinus* ticks in the current study was not sufficient to detect this bacterium.

Finally, no *Candidatus* N. mikurensis DNA was detected in either *I. ricinus* or *I. hexagonus* ticks feeding on *E. europaeus*. This finding is consistent with another study, in which this pathogen was also not detected in *I. hexagonus* feeding on Dutch hedgehogs [17]. Human and animal cases of *Candidatus* N. mikurensis infections have (as of yet) not been reported in the Netherlands, and the prevalence of this pathogen in questing *I. ricinus* ticks is relatively low [17]. Therefore, it is still unclear whether this pathogen may pose risk to public health in the Netherlands.

We detected *Salmonella* in 10% (9/90) of hedgehog faecal samples (Table 2). Salmonellosis is a zoonosis that has already been associated with hedgehogs, including *E. europaeus*. Several studies reported at least three different serotypes in hedgehogs that are pathogenic to humans: *Salmonella* Tilene, *Salmonella* Typhymurium and *Salmonella* Enteritidis [1,11]. Three isolates obtained from these faecal samples were characterized as *Salmonella enterica* subsp. *enterica* serotype Enteritidis, which is a common serotype pathogenic to humans [37].

**Table 2:** **Prevalence of enteric pathogenic bacteria, protozoan parasites, and ESC-resistant** ***E. coli*** **in hedgehog faeces**

	**April 2013**		**October 2013**		**Total**	
	**n = 58**	**%**	**n = 32**	**%**	**n = 90**	**%**
*Salmonella* spp.	1	2	8	25	9	10
*Campylobacter* spp.	0	0	1	3	1	1
Shiga toxin-producing *E.coli*	0	0	0	0	0	0
*Giardia* spp.	3	5	7	22	10	11
*Cryptosporidium* spp.	3	5	5	16	8	9
ESC-resistant *E. coli*	51	88	13	41	64	71
AmpC-producing *E. coli*	50	86	0	0	50	56
ESBL-producing *E. coli*	1	2	13	41	14	16

One faecal sample (1%) contained *Campylobacter*, which is the second most common food-borne bacterium worldwide [10]. Further genotyping revealed *C. jejuni*, which is recognized as one of the main causes of human gastroenteritis. A study in Denmark also reported the presence of *C. jejuni* in hedgehogs, which were rehabilitating in private homes, or fed in gardens [9]. No Shiga toxin-producing *E.coli* were detected in the faecal samples investigated.

In addition to zoonotic enteric bacteria, 11% (10/90) of hedgehog faecal samples were positive for *Giardia* species (Table 2). *Giardia* is a genus of flagellated protozoan parasites divided into eight major genetic groups (A-H) called assemblages, which slightly differ in morphology and may cause disease in diverse vertebrate hosts [13]. We detected *G. duodenalis* assemblage A in hedgehog faecal samples, which is responsible for human infections worldwide [12]. However, data regarding the presence of *Giardia* in hedgehogs are scarce, and until now no *Giardia* assemblages were found associated with these animals.

We detected *Cryptosporidium* species in 9% (8/90) of hedgehog faecal samples as well. *Cryptosporidium* is another genus of protozoan parasites of vertebrates, which causes enteric infections in humans. In this study, two genospecies, *C. parvum* (subtype: IIaA17G1R1 and IIcA5G3) and *C. hominis* (subtype: IbA10G2) were observed. These subtypes cause the majority of cryptosporidiosis in humans [14]. *Cryptosporidium hominis* subtype Ib is primarily transmitted anthroponotically and, to the best of our knowledge, it has never been detected in hedgehog faeces before. In addition, *C. parvum* subtype IIaA17G1R1 has never been detected in these animals either, but was described in calves, which may play a role in the transmission of human cryptosporidiosis [38]. Interestingly, subtype IIcA5G3, which is considered to be human specific, has been isolated from hedgehog faeces previously [7]. The presence of those subtypes in hedgehog faeces may indicate transmission of the pathogen within hedgehog populations, as suggested before [7].

Finally, viable ESC-resistant *E. coli* were detected in 71% (64/90) of hedgehog faecal samples (Table 2). AmpC-producing *E. coli* were only found in the samples collected in April, but at a high prevalence of 86% (50/58). A lower prevalence of 41% (13/32) of ESBL-producing *E.coli* was observed in samples collected in October. Only one isolate collected in April had the same phenotype. To the best of our knowledge this is the first description of ESC-resistant *E. coli* in hedgehogs. In the literature, an ESBL-producing isolate from hedgehog faeces was reported, however, this isolate was later identified as a *Klebsiella pneumoniaea* strain [39]. The relatively high prevalence of ESC-resistant *E. coli* in tested hedgehog faeces, especially in the April samples is intriguing. If this might pose a risk to humans, handling rehabilitating hedgehogs has to be investigated. It is very likely that these enteric pathogens, protozoan parasites, and ESC-resistant *E. coli* detected in hedgehog faecal samples were acquired by ingestion of contaminated materials found in the habitat of hedgehogs, and originates from other animals or humans (waste, food, etc.).

## Conclusions

Although hedgehog blood or tissue samples were not available for examination, a relatively high prevalence of vector-borne pathogens *B. burgdorferi* s.l. genospecies and *A. phagocytophilum* in engorged ticks obtained from *E. europaeus,* indicates that hedgehogs contribute to pathogen maintenance in natural cycles in (sub)urban areas in the Netherlands.

A number of enteric pathogenic bacteria, protozoan parasites, and ESC-resistant *E. coli* are present in faecal material, obtained from Dutch *E. europaeus.* This may pose a risk for people handling diseased and wounded animals, because they can come into contact with contaminated hedgehog faeces. However, to understand the transmission of infection between wildlife and humans, a thorough understanding of the population genetics of pathogens and hosts is required. To investigate this issue more in depth, isolates obtained from wildlife should be compared with human isolates, which represent serotypes that are epidemiologically important with regard to public health.
